# Genomic insights into cancer-associated aberrant CpG island hypermethylation

**DOI:** 10.1093/bfgp/els063

**Published:** 2013-01-21

**Authors:** Duncan Sproul, Richard R. Meehan

**Keywords:** epigenetics, epigenomics, cancer, DNA methylation, CpG islands

## Abstract

Carcinogenesis is thought to occur through a combination of mutational and epimutational events that disrupt key pathways regulating cellular growth and division. The DNA methylomes of cancer cells can exhibit two striking differences from normal cells; a global reduction of DNA methylation levels and the aberrant hypermethylation of some sequences, particularly CpG islands (CGIs). This aberrant hypermethylation is often invoked as a mechanism causing the transcriptional inactivation of tumour suppressor genes that directly drives the carcinogenic process. Here, we review our current understanding of this phenomenon, focusing on how global analysis of cancer methylomes indicates that most affected CGI genes are already silenced prior to aberrant hypermethylation during cancer development. We also discuss how genome-scale analyses of both normal and cancer cells have refined our understanding of the elusive mechanism(s) that may underpin aberrant CGI hypermethylation.

## INTRODUCTION

It is widely accepted that carcinogenesis necessitates multiple genetic alterations that either drive cellular division or remove checkpoints regulating this process in normal cells. These same disruptions could potentially also be caused by epimutations. Epigenetic events have been strictly defined as heritable changes in gene function that are not explained by changes in DNA sequence but also more recently as ‘the structural adaptation of chromosomal regions so as to register, signal or perpetuate altered activity states’ [1]. Here, we refer to epimutations as heritable, abnormal alterations in the state of chromosomal regions.

Strong support for the possibility that epimutations play a significant role in cancer comes from the recent discovery that epigenetic regulators are recurrently mutated in cancer genomes and observations that the levels and distributions of epigenetic marks are altered in cancer [2]. Particular attention and research effort has focused on the hypothesis that aberrant silencing of genes by DNA hypermethylation is a key epimutational mechanism driving carcinogenesis. Importantly, a clear mechanism has been described for the inheritance of DNA methylation patterns across cellular generations [1] and, therefore, abnormal DNA methylation states fit the definition of epimutations as heritable alterations.

**Figure 1:**
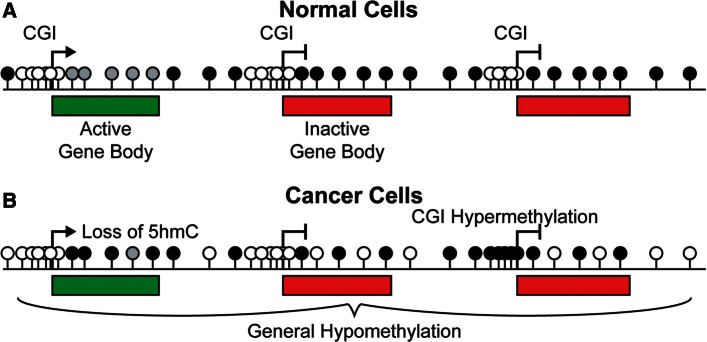
The methylation landscape of normal and cancerous cells. (**A**) The mammalian genome is depleted of CpGs and the majority of these are methylated (black lollipops). CGIs are rich in CpGs, frequently coincide with gene promoters and are generally unmethylated (white lollipops), irrespective of gene expression status. The bodies of active genes are enriched in hydroxymethylated CpGs (grey lollipops). (**B**) Both DNA methylation and hydroxymethylation are reduced in cancer genomes but some CGIs become aberrantly hypermethylated.

In normal mammalian somatic genomes, DNA methylation mainly occurs at cytosines in a CpG dinucleotide context [3]. Around 70% of CpGs in mammalian genomes are methylated but DNA methylation is bimodally distributed and is generally absent from short stretches of CpG-rich sequence known as CpG islands (CGIs) [4] which frequently correspond to the promoters of genes [5] (Figure 1A). The remainder of the genome is relatively depleted in CpGs due to the inherent mutability of methylcytosine (mC) which is prone to spontaneous deamination [6]. Such deamination can also cause cancer-associated mutations, including many *TP53* (p53) mutations [7]. Methylated cytosines in the genome are recognised by methyl CpG binding proteins (MBDs) which are hypothesised to play an important role in reading this epigenetic mark [8]. During embryogenesis, the mammalian genome undergoes a series of epigenetic reprogramming events including a wave of global demethylation followed by establishment of the bimodal pattern by the *de novo* methyltransferases DNMT3A and DNMT3B [9]. The maintenance DNA methyltransferase, DNMT1, ensures that this distribution is stably inherited during the remainder of development and differences in the DNA methylation profiles between adult cell types have been shown to be relatively small, particularly at CGIs which are generally maintained in a hypomethylated state regardless of gene expression status [10, 11]. Regions bordering CGIs in the human genome, termed CGI shores, have been suggested to be more variably methylated between different normal cell types [12, 13]. At present, however, it is unclear whether they represent a distinct functional genomic compartment. Hydroxymethylation of cytosines (hmC) has also been recently rediscovered in mammalian cells [14] and significant levels of this modification are present in the bodies of active genes in some somatic tissues [15].

**Figure 2:**
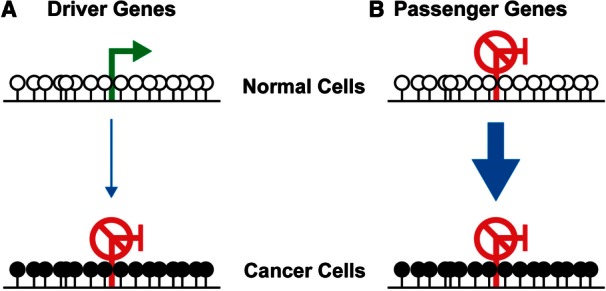
Hypermethylation of driver and passenger genes in carcinogenesis. (**A**) Aberrant hypermethylation of CGIs is thought to cause the silencing of tumour suppressor genes and drive carcinogenesis. (**B**) The analysis of cancer methylomes demonstrates the majority of hypermethylated genes are repressed in preneoplastic cells. Hypermethylation of these, passenger, genes might be a surrogate of general epigenetic dysfunction that occasionally results in hypermethylation and repression of active driver genes. White lollipops - unmethylated CpGs, black lollipops - methylated CpGs.

In cancer cells, genomic levels of DNA methylation are frequently reduced compared with their normal counterparts [16]. The underlying cause(s) of this reduction is unknown, but the loss can be localised to particular types of repetitive elements [17] or chromosomal domains [18, 19] (Figure 1B). Global levels of hydroxymethylation have also recently been shown to be reduced in cancer cells [15, 20–22]. Contrasting with this overall trend, many CGIs undergo cancer-associated aberrant hypermethylation [18] (Figure 1B). Hypermethylation of CGI promoters is tightly linked with transcriptional repression of the affected gene [23] and many promoters initially shown to be aberrantly hypermethylated in cancer correspond to known tumour suppressor genes (TSGs) [24]. Aberrant CGI hypermethylation has, therefore, been viewed as an epimutation causing the silencing of TSGs (Figure 2A) [25]. Thus, a strong hypothesis is that the aberrant hypermethylation of CGIs can drive carcinogenesis and cancer progression and that epimutational events might outnumber mutations in cancer [26].

Here, we review our current understanding of aberrant CGI hypermethylation as a paradigm of a potential epimutation in cancer. In particular, we focus on how recent data from the study of cancer methylomes demonstrates that the majority of genes that are aberrantly hypermethylated in cancer are in fact already repressed in preneoplastic cells. This parallels the view that most CGI hypermethylation in normal development occurs subsequent to silencing by other means. Based on these findings, we present alternative hypotheses as to the impact of aberrant CGI hypermethylation on the growth and development of cancers. Finally, we describe how the genome-scale analysis of cancer has advanced our understanding of the potential molecular mechanisms behind this epigenetic reprogramming.

## EVIDENCE FOR INACTIVATION OF TUMOUR SUPPRESSOR GENES BY ABERRANT HYPERMETHYLATION

Evidence that aberrant CGI hypermethylation might act as an epimutation directly driving carcinogenesis is based primarily on studies of individual candidate genes. The aberrant hypermethylation of genes such as *RB1* [27–30], *MLH1* [31, 32] and *BRCA1* [33], whose mutation is associated with inherited cancer predisposition [34], can be regarded as particularly significant. Three important pieces of evidence support the view that aberrant hypermethylation in cancer causes their silencing.

First, hypermethylation of TSGs has been observed alongside inherited germline mutations [35–37]. This suggests promoter hypermethylation can directly substitute for genetic loss of heterozygosity (LOH) as the second hit that completely disable TSG activity. The evidence is particularly strong in the case of *CDNK2A* (p16/ARF) where the presence of a mutation in the first exon in a colon cancer cell line facilitated the direct demonstration that hypermethylation occurs only on the wild-type allele [36]. In the majority of cases, however, such analyses have not or cannot be conducted and it remains to be demonstrated whether hypermethylation frequently occurs in an allele-specific fashion. Recent studies of *BRCA1* have failed to observe instances of LOH through hypermethylation, suggesting that it might be a very rare event [38, 39].

Second, the tissue specificity of TSG hypermethylation in sporadic cancer overlaps with the tissue-specific predispositions caused by inherited mutations in these same genes. Inherited *MLH1* mutations predispose to colorectal cancer and *MLH1* hypermethylation is largely limited to colorectal tumours [40]. Similarly, *BRCA1* mutations predispose specifically to breast and ovarian cancer and hypermethylation is limited to cancer of these tissues [40]. Within particular tissues, the phenotypes of cancers which have hypermethylated particular TSGs can overlap with the specific phenotypes of cases associated with inherited mutations in the same gene. For example, *RB1* mutated and hypermethylated retinoblastomas phenocopy each other [37], colorectal tumours with either mutated or hypermethylated *MLH1* have microsatellite instability (MSI) [32] and *VHL* hypermethylation occurs in renal cancers of the clear cell histological subtype as do *VHL* mutations [41]. Rare cases of inherited methylation of *MLH1* and *MSH2* also confer a predisposition to developing MSI colorectal cancer as do mutations in these genes, although these apparent epimutations are tightly associated with genetic variants [42, 43]. The overlap between the phenotype of mutated and hypermethylated cancers is less clear in other cases. Carriers of *BRCA1* mutations develop particular types of breast cancer, specifically estrogen receptor negative (ER−ve) tumours that are often classified as belonging to one of a few special histological types [44]. *BRCA1* hypermethylation was reported to be more frequent in medullary carcinomas which are ER−ve and often occur in *BRCA1* carriers, but is also observed in mucinous carcinomas which are ER+ve [33, 45, 46]. Subsequent studies have reported that *BRCA1* hypermethylation is not specific to ER−ve breast cancers [47] and that the gene expression profiles of *BRCA1* mutated and methylated cancers differ [48]. *BRCA1* hypermethylated serous ovarian adenocarcinomas also displayed a clinical course that was more similar to *BRCA1* wild type than *BRCA1* mutant tumours [49]. It should be noted, however, that inherited and somatic mutations in important TSGs can be associated with different phenotypes. For example, inherited mutations in *CDNK2A* predispose to melanoma and pancreatic tumours [34] but somatic *CDNK2A* mutations occur in a variety of other cancer types including non-small cell lung cancer [50].

The final strong piece of evidence underpinning the causative role of aberrant DNA hypermethylation in silencing tumour suppressor genes in cancer is that they can be reactivated when methylation is removed from their promoters. This is most often achieved by treatment with the drug 5′-aza-2′-deoxycytidine (5-Aza) which causes the degradation of DNMT1, the maintenance methyltransfrase [51]. Treatment of cancer cell lines with 5-Aza causes the reactivation of hypermethylated *VHL* [41], *MLH1* [32, 52] and *BRCA1* [53]. Also, genetic knockout or RNA-mediated knockdown of DNMT results in activation of previously hypermethylated *CDNK2A* in a colorectal cancer cell line [54, 55]. These experiments, however, do not examine the temporal sequence of events causing gene silencing. Treatment of female mammalian cells with 5-Aza causes the activation of genes on the inactive X chromosome (Xi) [56], but silencing of genes on the Xi precedes hypermethylation of gene promoters [57, 58] and can occur in the absence of DNMTs [59, 60]. These observations demonstrate that gene activation can occur upon ablation of promoter hypermethylation even when the hypermethylation was not the initial and causative silencing event.

## MOST ABERRANTLY HYPERMETHYLATED CGI GENES ARE REPRESSED PRIOR TO HYPERMETHYLATION

The evidence for aberrant CGI hypermethylation as a direct silencer of TSGs is mostly correlative leading many to question its direct role in carcinogenesis [61–64]. Although cancer-associated hypermethylation of a gene’s promoter has been invoked to suggest it might possess tumour suppressor activity [65], many aberrantly hypermethylated genes are unlikely candidates as TSGs, for example, *CALCA* (Calcitonin), the first gene reported to become hypermethylated in cancer [66]. Early unbiased studies of cancer methylomes made it clear that large numbers of genes could be hypermethylated in a single specimen [67]. The most dramatic cases are cancers with CGI hypermethylator phentotypes (CIMP), first described in colorectal tumours [68] and more recently in cancers arising in other tissues [69–72]. The frequency of aberrant hypermethylation has been used to suggest that epimutations might be more significant than mutations in carcinogenesis [26], but this is difficult to reconcile with evidence suggesting that relatively few mutations are likely to be required for carcinogenesis [34, 73].

In order for aberrant hypermethylation to directly drive cancer by silencing genes, the affected genes must be expressed prior to hypermethylation. Transcriptionally repressed genes have been known to undergo hypermethylation in tissue culture for many years [74]. Recent integrated analyses of cancer methylomes together with gene expression data demonstrate that transcriptionally repressed genes are in fact also the predominant target of cancer-associated aberrant hypermethylation (Figure 2B). By analysing the methylation profiles of cancers derived from seven different tissue types, we have shown that genes which are repressed in a lineage-specific fashion in normal tissues become hypermethylated in cancers derived from that tissue, whereas housekeeping or expressed lineage-specific genes are resistant to hypermethylation [40, 75]. A study of colon cancer found that 93% of the genes hypermethylated in CIMP tumours had unaltered expression in tumours compared with normal tissue [76]. This suggests they are already repressed in the normal colon because CGI hypermethylation is tightly associated with transcriptional repression. One explanation for the low correlation frequently observed between gene expression changes and promoter hypermethylation in cancer methylomes studies is that the majority of affected genes are normally repressed in the tissue studied [71, 77–81]. A comparison of an osteosarcoma cell line to cultured mesenchymal stem cells and osteoblasts also found that the majority of aberrantly hypermethylated genes in the osteosarcoma cell line were repressed in the normal cells analysed [82]. It is possible that hypermethylation prone genes are expressed to a low level rather than repressed in normal tissue [18] but background levels of hybridisation to probes make it difficult to draw this conclusion from microarray expression data. A recent and comprehensive analysis of the normal expression of *RUNX3*, which is frequently hypermethylated in gastric cancers, conclusively demonstrated that it is in fact never expressed in the cells that give rise to these tumours and supports the hypothesis that hypermethylation prone genes are fully repressed rather than expressed to a low level [65].

These recent findings from the study of cancer methylomes draw parallels with our understanding of CGI hypermethylation during normal development which, most evidence suggests, occurs at genes already repressed through other mechanisms [83, 84]. As noted above, the hypermethylation of CGIs on the Xi in female cells occurs after genes have already been silenced [57, 58]. Mice deficient for the *de novo* methyltransferases initiate X-inactivation normally [60] and genetic deletion of *Dnmt1* does not preclude X-inactivation but instead results in sporadic reactivation of the Xi later in development [59]. Repression of the paternal copy of the imprinted *Meg3* (*Gtl2*) promoter in mouse development occurs prior to its hypermethylation [85]. The CGI promoter of the pluripotency associated transcription factor *Oct-3/4* is also silenced before becoming hypermethylated during differentiation and functional studies implicate hypermethylation in stabilising its silencing [86, 87]. It is repressed gene promoters that become hypermethylated during the *in vitro* differentiation of mouse ES cells [88] and silencing of a transgene in chicken erythroid cells precedes its hypermethylation [89]. A stabilising role for CGI hypermethylation is also supported by observations that DNA methylation represents a barrier to the reprogramming of somatic cell types to induced pluripotent stem cells (iPS cells) [90] and that its ablation increases reprogramming efficiency [91]. Taken together, findings from the study of cancer methylomes put cancer-associated aberrant CGI hypermethylation in a similar framework to CGI hypermethylation in normal development, as a largely secondary event (Figure 2B).

## DO EPIGENETIC EVENTS IN CANCER FOLLOW A DRIVER AND PASSENGER MODEL?

Although the analysis of cancer methylomes demonstrates that the vast majority of genes whose CGI promoters become hypermethylated in cancer are repressed prior to hypermethylation, the possibility remains that occasionally active genes become hypermethylated and repressed (Figure 2). Such genes have been termed epigenetic drivers [92, 93] and the bulk of hypermethylated genes, which are repressed in normal untransformed tissue, are hence termed passengers, a nomenclature adopted from studies of the mutational structure of cancer genomes [94]. The integrative analyses described above do not exclude the possibility that a small proportion of hypermethylated genes might be active in the tissue of origin [40, 76] and one study has suggested that a significant proportion of genes hypermethylated in an osteosarcoma cell line are active in normal cells [82]. The estimation of the exact proportion of normally active genes that are aberrantly hypermethylated in cancer is, however, complicated by a number of issues. First, normal tissues consist of a heterogenous mix of cell types and cancers often originate from rare cell populations, and therefore, the bulk expression profile of a normal tissue might not be representative of a cancer's cell of origin [95]. Second, in these analyses, one measurement of expression level is generally used for the whole gene. The existence of alternative promoters, however, means that hypermethylation of an apparently active gene may actually occur at an inactive promoter, as has been described for *APC* in gastric cancer [96]. Clarification of this situation will require the analysis of rigorously purified cell populations using techniques that measure promoter transcriptional activity, such as CAGE [97], and carefully constructed bioinformatic analysis pipelines which focus on promoters rather than whole genes.

The strong phenotype of cancer predisposition genes which are also hypermethylated [32, 33] demonstrates that they must be expressed in the affected normal tissues. A key question in these cases is whether their aberrant hypermethylation is also secondary? The potential primary repressive event would have to occur abnormally rather than as part of normal development, as is the case for the majority of aberrantly hypermethylated genes. *MLH1* is frequently hypermethylated in colorectal tumours with a CIMP phenotype [76] supporting the possibility that *MLH1* becomes hypermethylated through the same mechanism affecting passenger genes. *BRCA1* is repressed in sporadic breast cancer in the absence of hypermethylation [47] suggesting that it too could be repressed prior to hypermethylation. Silencing of *BRCA1* in cell lines can be initiated by the transcription factors SNAI1 and SNAI2 (Snail and Slug, respectively) in partnership with the histone lysine demethylase LSD1 [98]. An interesting example is *CDKN2A* which is frequently hypermethylated in a variety of tumour types [99]. *CDKN2A* is normally only expressed in cells following replicative or oncogenic stress and it remains silent in most normal cells [100]. This suggests the possibility that the gene could become hypermethylated prior to transformation. *CDKN2A* is normally repressed by polycomb repressive complexes (PRCs) [101, 102] which have been implicated as part of the mechanism associated with aberrant hypermethylation (see below). Furthermore, after reactivation following genetic knockout of *DNMT1* and *DNMT3B* in a colon cancer cell line, *CDKN2A* is silenced before DNA hypermethylation is re-established [103] and *CDNK2A* silencing also occurs prior to hypermethylation in colonies of human mammary epithelial cells (HMECs) that escape replicative arrest in culture [104].

Taken together, these observations suggest that although some hypermethylated genes might be expressed in normal tissues, their hypermethylation could follow aberrant down-regulation by other means. Another possibility is that separate mechanisms result in the hypermethylation of active and repressed genes. A recent study demonstrated that the hypermethylation of initially active genes on the Xi requires additional factors compared with initially silent genes supporting the idea that alternative mechanisms might underpin the hypermethylation of different gene types [105]. Non-coding genetic variants may also cause aberrant CGI hypermethylation, as exemplified by cases of inherited allelic hypermethylation of *MLH1* and *MSH2* which are tightly associated with sequence variants [42, 43]. The case of *CDNK2A* suggests that prior expression in normal tissue is not a perquisite for the hypermethylation of a gene playing a role in promoting carcinogenesis. Also, even in cases of driver genes where hypermethylation was not the initiating silencing event, its role in maintaining silencing might be important for the continued growth of the cancer. Potential epigenetic driver genes have been identified by screening for genes whose promoters remain aberrantly hypermethylated after genetic ablation of DNA methyltransferase activity in a hypomorphic colorectal cancer cell line [106]. The identified genes were not classically known to be TSGs and their expression in normal colon was not demonstrated but their enforced expression via a strong CMV promoter in wild-type colon cancer cell lines inhibited their growth suggesting that maintenance of hypermethylation at these genes was important for the fitness of the cell line.

While the potential that the hypermethylation of some genes is subject to positive selection because it facilitates carcinogenesis fits current data from methylome studies, a number of questions remain. One prediction of the driver and passenger model is that tumours with methylator phenotypes would demonstrate more aggressive clinical behaviour because they would be statistically more likely to have hypermethylated more TSGs or drivers. Methylator phenotypes in colorectal tumours, breast tumours and glioblastomas, however, all coincide with better clinical prognoses [69, 71, 107]. It also remains to be explained why the mutational and hypermethylation landscapes are so different if the selection of rare driver events underpins their development. Putative passengers, that is to say the majority of aberrantly hypermethylated genes, are frequently and reproducibly hypermethylated in cancer whereas the strongest candidates for driver genes, those that confer cancer predisposition when mutated, are hypermethylated much more rarely [40]. This contrasts with the mutational landscape of cancer where individual driver genes are observed to be more frequently mutated in clinical samples than passenger genes as a result of selection [108].

## ALTERNATIVE HYPOTHESES AS TO THE ROLE OF ABERRANT CGI HYPERMETHYLATION IN CANCER

Rather than playing an evolutionary neutral passenger role and representing a surrogate of general epigenetic dysregulation, the widespread hypermethylation of normally repressed genes in cancer could have other impacts on carcinogenesis and progression. As discussed above, hypermethylation of CGIs in normal development results in stable gene silencing and prevents ectopic activation [83]. It is conceivable that abundant promoter hypermethylation could influence the epigenetic plasticity of cancer cells with two possible outcomes.

**Figure 3:**
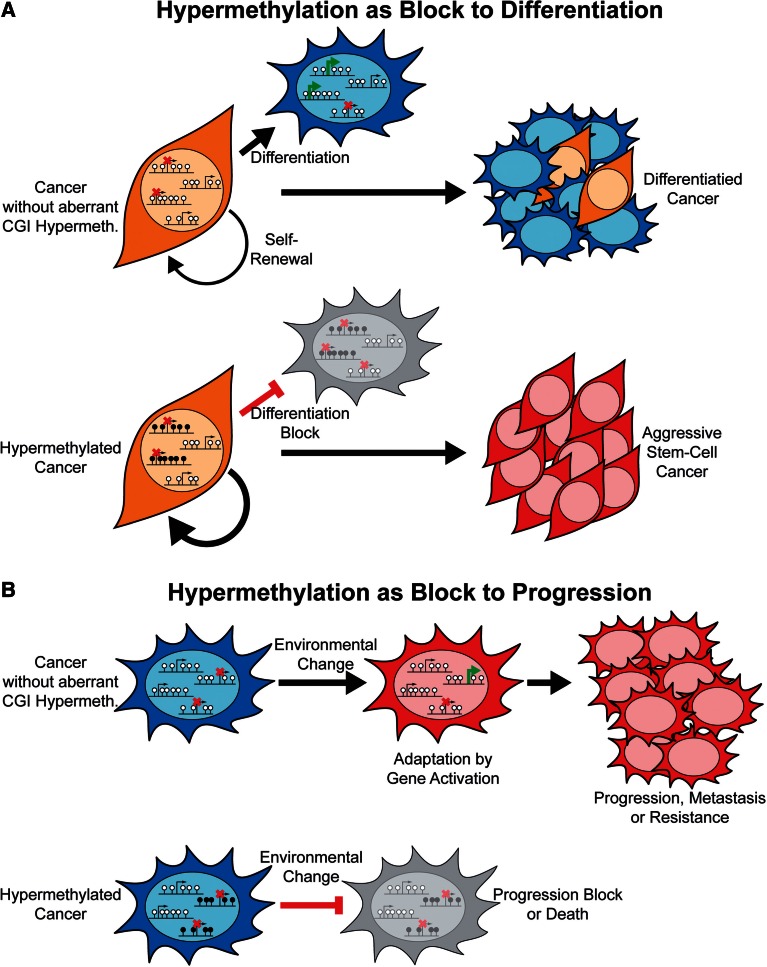
Models as to the consequences of widespread CGI hypermethylation in cancer. (**A**) Hypermethylation as a block to differentiation. This model predicts that key genes required for normal cellular differentiation become hypermethylated in cancer. The result is a block to their activation and thus normal differentiation processes within the cancer producing a more aggressive, stem-cell like phenotype. (**B**) Hypermethylation as a block to progression. Widespread hypermethylation of repressed CGI promoters might prevent their stochastic activation in individual cancer cells. If the activation of such genes facilitated survival in changing conditions, such as during metastasis to distant organs or treatment, widespread hypermethylation might restrict the potential for epigenetic adaptation and thus result in block to progression.

Many groups have noted that genes repressed by PRCs in embryonic stem (ES) cells are frequently hypermethylated in cancer [109–111]. Other studies have reported overlaps in the gene expression profiles of ES cells and aggressive cancers [112] which, at least in part, correspond to repression of ES cell PRC targets in cancers [113]. Many of the genes targeted by PRCs in ES cells are transcription factors whose expression is key to lineage commitment during differentiation [114]. Thus, it has been proposed that the frequent hypermethylation of ES cell PRC targets in cancer might block differentiation and maintain cancers in a stem-cell-like state [111] (Figure 3A). In support of this hypothesis, genes which are expressed late in murine-lung differentiation are reported to be frequently hypermethylated in non-small cell lung cancer [115]. *IDH1 R132H* mutations correlate with a hypermethylator phenotype in acute myeloid leukaemia (AML) [70] and knock-in of this mutant into mouse haematopoietic stem cells results in general hypermethylation and an apparent block to differentiation [116]. These mutations, however, result in the production of an oncometabolite, 2-hydroxygluterate, which affects the function of numerous cellular enzymes [117, 118] and the differentiation block observed in this study is not necessarily a result of CGI hypermethylation. The hypothesis that widespread hypermethylation maintains cancers in a aggressive stem-cell-like state is also inconsistent with the better clinical prognoses associated with cancer hypermethylator phenotypes [69, 71, 107].

Instead of maintaining a stem-cell-like state, a restriction of epigenetic plasticity induced by widespread hypermethylation could act as a check on cancer progression (Figure 3B). The dissemination of cancer cells from the site of origin and their survival in metastatic niches requires the activation of gene expression programs [119]. Resistance to therapy can occur as a result of secondary activating mutations [120, 121] and potentially through epigenetic gene activation. The stable gene repression associated with hypermethylation might provide a barrier to these events resulting in an inhibition of cancer progression. This hypothesis is more consistent with the better prognosis associated with hypermethylator phenotypes. Genes whose expression is associated with metastasis are hypermethylated as part of a breast cancer hypermethylator phenotype [69]. One potential prediction of a hypermethylation protective model is that aberrant CGI hypermethylation might occur as part of a cellular defence mechanism. Intriguingly, cellular senescence is associated with epigenetic alterations but it is unclear at present whether these include the widespread hypermethylation of CGIs [122]. If a cellular defence mechanism does result in aberrant CGI hypermethylation, it provides an alternative explanation for the observation of CGI hypermethylation in pre-cancerous lesions which is commonly presented as evidence of the importance of epimutations in the earliest stages of carcinogenesis [123].

Cancers are, however, very heterogenous with respect to their genetic and epigenetic profile and the environment in which they grow; the two hypotheses outlined above may, therefore, reflect differing roles of widespread aberrant hypermethylation in different cancer types or subtypes. They both may even play a part in the same cancer at different stages of progression. For example, a hypermethylation-mediated block on differentiation may promote the initial growth of a tumour but later this restricted epigenetic landscape might prevent its metastasis. Furthermore, neither of these hypotheses is incompatible with the occasional hypermethylation of driver genes outlined above. Differentiating these possibilities requires an understanding of the mechanism responsible for aberrant CGI hypermethylation.

## THE MECHANISM(S) OF ABERRANT CGI HYPERMETHYLATION IN CANCER

Although the mechanism(s) responsible for aberrant promoter hypermethylation in cancer remains elusive, potential hypotheses have emerged from genome-scale analyses of both normal and cancerous cells. Two main types of mechanisms have been proposed; active processes mediated by targeting of specific factors to CGIs or passive mechanisms resulting from a loss of protection against *de novo* methylation.

One hypothesis is that aberrant CGI hypermethylation in cancer results from the over-expression or increased activity of DNMTs. Such increases were initially reported [124, 125] but are likely to be attributed to the regulation of DNMTs during the cell cycle [126, 127] and an increased number of cycling cells in cancer. A recent analysis reports that hypermethylation at some genes correlates with increased DNTM3B levels in colorectal tumours [128]. Experimental manipulation of DNMT levels in the *Apc^min/+^* mouse model of colorectal cancer demonstrate that higher DNMT levels promote carcinogenesis [129–131]. *Dnmt3b* over-expression in this model is also associated with promoter hypermethylation of the murine homologues of genes hypermethylated in human colorectal tumours [132]. On the other hand, *DNMT3A* mutations occur in AML and other haematological cancer genomes [133, 134] and these mutations have been shown to reduce DNMT3A enzymatic activity [135]. They have not, however, been found to correlate with variations in CGI hypermethylation patterns [133, 136]. *DNMT3B* mutations in cancer have not currently been found but aberrant splicing of *DNMT3B* frequently gives rise to cancer-specific isoforms of the enzyme [137]. These isoforms lack a methyltransferase domain but they could potentially function similarly to DNMT3L, acting as cofactors to bring canonical DNMT3A and 3B to new locations and stimulating their activity [138, 139]. Although variations in DNMT activity might affect carcinogenesis, it remains to be demonstrated whether this occurs as a result of increased CGI hypermethylation. It is also unclear as to whether MBDs play any role in the process of aberrant CGI hypermethylation. The MBDs *Mbd2* and *Kaiso* appear have contributary roles in intenstinal tumorogenesis as their deletion in mice result in reduced tumour numbers in *Apc^min/+^* mice [140, 141]. As with the DNMT work described above, however, these phenotypes have not been shown to be connected to aberrant CGI hypermethylation and currently no mutations in MBDs have been described in cancer.

As noted above, genes marked by PRCs in ES cells are frequently hypermethylated in cancer. DNMT3A and 3B biochemically interact with EZH2, a member of the PRC2 complex [142], leading to the suggestion that PRCs might recruit DNMTs to aberrantly hypermethylated genes. This interaction has, however, been reported to be cell type-specific [143] and artificial recruitment of EZH2 to a genomic locus does not result in hypermethylation [144]. DNA methylation and the PRC-associated histone mark, H3K27me3, rarely overlap at gene promoters [145, 146] and direct bisulfite sequencing of material from chromatin immunoprecipitation (ChIP) demonstrates that H3K27me3 and DNA methylation do not co-occur at CGIs [147, 148]. Recent evidence suggests that DNA methylation may restrict PRC distribution [148, 149], at least in ES cells, but there is currently no direct evidence to suggest the opposite scenario; i.e. CGIs are protected from hypermethylation by PRC occupancy. H3K27me3-marked CGIs that become hypermethylated in cancer have, however, been reported to lose this histone mark [150].

Histones and their associated marks may play other roles in protecting CGIs from hypermethylation. In normal cells, the histone mark H3K4me3 is anti-correlated with DNA methylation [151]. H3K4me3 is intimately associated with CGIs due to the presence of Cfp1 which recruits Set1, a H3K4 methylase [152]. DNMT activity during early development is directly inhibited by H3K4me3 because DNMT3L cannot bind histones carrying this mark [153] but *Cfp1* knockout in mouse ES cells does not result in CGI hypermethylation [154]. The variant histone H2A.Z is also anti-correlated with DNA methylation in plants [155], fish [156] and human cells [157]. Mutation of the enzyme responsible for H2A.Z deposition in plants results in gains of methylation [155] but the mechanistic basis for this relationship however remains unknown and no somatic defects in H2A.Z have been reported in cancer.

The analysis of cancer methylomes has also linked dysfunction in DNA demethylation pathways to aberrant CGI hypermethylation. DNA demethylation is proposed to be initiated by the conversion of mC to hmC by the ten-eleven translocation (TET) family of enzymes [158, 159]. Tet1 is bound to CGIs in mouse ES cells [160, 161] leading to the proposal that it maintains the fidelity of DNA methylation patterns in cells by maintaining CGIs in a hypomethylated state [162]. As noted above, reduction in global hmC is frequent in cancer [15, 20–22] and disruptions to TET enzyme function have been linked to CGI hypermethylation. Mutations abrogating TET2 enyzmatic activity are frequent in AML [163] and correlate with a hypermethylator phenotype [70]. The oncometabolite 2-hydoxygluterate is also produced in AMLs with *IDH1* or *2* mutations and one of its effects is the inhibition of TET enzyme activity [118]. *IDH* mutations in AML correlate with a similar hypermethylator phenotype to *TET2* mutations [70] and CIMP in glioblastoma is also associated with *IDH1* mutations [71]. Although one study suggested *Tet1* knockdown in mouse ES cells led to CGI hypermethylation [161], the same observation was not made in a similar independent study [160]. As TET1 is also bound to the vast majority of CGIs in mouse ES cells, it is unclear why TET dysfunction in cancer might result in the preferential hypermethylation of repressed CGIs.

Epigenomic analyses of normal cells point towards DNA sequence determining genome methylation patterns through sequence-specific transcription factors (TFs) rather than *vice versa* [164–166]. DNMT3A and 3B have been shown to interact with normal TFs [167] and abnormal versions generated as a result of gene fusions [168]. Although a number of studies have published sequence motifs associated with genes that become hypermethylated in cancer, these have neither been reproduced nor demonstrated to correspond to the binding sites of particular TFs [78, 169]. As TFs are found at both active and repressed genes, it is unclear why their recruitment of DNMT would specifically result in the hypermethylation of repressed genes. A reproducible association does occur between general TF motifs and hypermethylation-resistant promoters [18, 170] which is consistent with their housekeeping gene status [40]. In addition to suggesting that TF binding protects CGIs from hypermethylation, this correlation could be explained if transcriptional activity itself conferred protection from hypermethylation. One model for the generation of bimodal hypermethylation patterns in mammalian cells postulates that CGIs are protected from *de novo* methylation by transcription during the developmental window in which the genome is remethylated [63]. An analysis comparing normal and cancer cell lines also showed that the presence of stalled or active RNA polymerase in normal cells predicts resistance to aberrant hypermethylation in cancer cells [171]. Variations in DNA sequence that alter promoter activity also correlate with the predisposition of promoters to hypermethylation. An extra SP1 site in the *RIL* gene confers resistance to hypermethylation [172] and sequence variants in the *MLH1* promoter that reduce promoter activity are associated with soma-wide mosaic hypermethylation [42]. It is unclear, however, if low-level transcription confers resistance to CGI hypermethylation or whether the relationship is quantitative with increasing transcription levels resulting in a lower frequency of hypermethylation but not entirely excluding it. Models of protection based on the hypothesis that transcription protects CGIs must also consider the presence of stalled RNA polymerase at PRC-marked genes [173, 174].

One of the main results to emerge from the systematic analysis of cancer methylomes is the finding that cancer-associated hypermethylation does not occur at random but affects a distinct set of genes. As noted above, several groups have documented the overlap between PRC-marked genes in ES cells and hypermethylation in cancer and this has been reproduced extensively in genome profiling studies [76, 109–111, 175]. Rather than being repressed, PRC-occupied CGIs in ES cells are proposed to exist in a poised state [176] which is resolved to either full activation or repression as differentiation proceeds [177]. A subset of PRC-marked genes in ES cells are, therefore, expected to be occupied by PRCs in differentiated cell types. The association between PRC-marked CGIs in ES cells and hypermethylation in cancer might, therefore, reflect preferential hypermethylation of those repressed CGIs associated with PRCs in adult cells during the transformation process rather than a mechanistic connection between ES cell and cancer epigenetic state. The promoters of hypermethylation-prone genes are also relatively depleted of retrotransposons compared with hypermethylation-resistant promoters [178]. This could result from evolutionary selection against retrotransposon integration near tissue-specific genes because it might disrupt essential interactions with distal regulatory elements [40]. Overall, the characteristics of hypermethylation-prone genes are consistent with the possibility that repressed, lineage-specific genes are predominantly subject to cancer-associated hypermethylation [40]. While the observation that aberrant hypermethylation affects a specific set of genes has been presented as evidence for a targeted rather than stochastic process of hypermethylation [179], it does not exclude the possibility that stochastic hypermethylation occurs with a susceptible set of genes. One recent study has suggested that normal cell line promoter methylation patterns evolve through a stochastic process [180].

**Figure 4:**
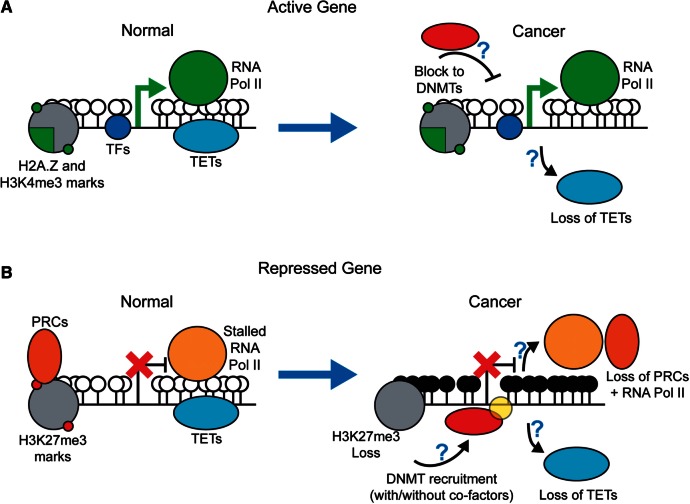
Potential mechanisms of cancer-associated aberrant hypermethylation. CGIs bound are occupied by multiple factors that potentially play roles in aberrant CGI hypermethylation. (**A**) Nucleosomes incorporating the histone variant H2A.Z and with H3K4me3 marks, TFs, active transcription and TET enzymes may all help maintain the hypomethylated state of active CGIs in normal cells. TET enzyme function is most likely compromised in at least some cancers but this still leaves other protective factors at active CGIs. (**B**) Similarly, a number of factors are found at inactive CGIs that could potentially play a role in maintaining their normal hypomethylated state including TET enzymes, PRCs and stalled RNA polymerase. PRCs are thought to be lost, most likely along with stalled RNA polymerase, when CGIs become aberrantly hypermethylated, but it is unclear if this plays a mechanistic role in this cancer-associated epigenetic reprogramming. The role of DNMT recruitment in the process is also unclear. White lollipops - unmethylated CpGs, black lollipops - methylated CpGs.

Taken together, these studies suggest that many factors could be involved in the reprogramming of repressed CGIs to a hypermethylated state in cancer. It is clear that a number of different factors found at active CGI promoters are capable of maintaining them in a hypomethylated state including H3K4me3, H2A.Z, TFs, TET enzymes and active RNA transcription (Figure 4A). Repressed CGIs are also occupied by a number of factors that could potentially perform the same function including TETs, PRCs and RNA polymerase (Figure 4B). Furthermore, some of these factors are shared with active CGIs, so it is uncertain why a defect in any one factor would result in aberrant CGI hypermethylation (Figure 4B). The potential role of active recruitment of DNMTs in this picture is also unclear and the relative importance of stochastic and targeted processes in the evolution of cancer CGI methylomes remains to be determined.

## SUMMARY AND CONCLUSIONS

The recent characterisation of cancer methylomes has demonstrated, contrary to the prevailing view, that the hypermethylation of CGI promoters in cancer parallels CGI hypermethylation during normal development and is secondary to silencing by other means. For most aberrantly hypermethylated promoters, this silencing occurs as a result of normal development and subsequent methylation represents an epigenetic reprogramming event. Potential driver genes that are expressed in normal cells could conceivably be directly silenced by aberrant hypermethylation. The overwhelming tendency for hypermethylation to occur as a secondary event, however, strongly suggests that such drivers are subject to primary aberrant silencing in cancer through other means. Hypermethylation of isolated individual repressed genes may also contribute a growth advantage to cancer by preventing their activation at later stages of carcinogenesis or progression. We should also consider whether the widespread hypermethylation of CGIs in cancer has other impacts on the growth of cancers, in particular by blocking differentiation or restricting epigenetic plasticity and adaptive potential.

The analysis of cancer genomes and methylomes have helped refine our definition of the type of gene affected by aberrant promoter hypermethylation and generated new hypotheses as to the molecular defect underpinning this epigenetic reprogramming but many questions remain to be answered. Dissection of this mechanism is also likely to lead to new insights regarding the biology of CGIs, the most abundant promoter type in our genome [63]. The reinterpretation of cancer-associated CGI hypermethylation that has occurred as a result of the advent of genome-scale datasets should also be considered as potential epimutations associated with other diseases are identified [181]. Finally, cancer epigenomes are potentially a rich source of biomarkers and specific epigenetic defects may be exploitable therapeutic targets [182]. We have not discussed these avenues of research here, but our new more global understanding of cancer-associated CGI hypermethylation should be used to guide these efforts.

As this review was going to press, de-repression of *CXCR4*, which has a CGI promoter, was shown to facilitate metastasis in a renal cancer cell line [183]. This further supports our proposal that widespread aberrant hypermethylation of CGI promoters in cancer could inhibit progression by blocking gene activation (Figure 3).

**Key Points**

- CpG islands (CGIs) frequently become aberrantly hypermethylated in cancers.
- Analysis of cancer methylomes has shown that aberrant CGI hypermethylation occurs primarily at genes that are already silent in the host tissue and is therefore not generally linked to transcriptional silencing of tumour suppressor genes.
- The predominant view is that CGI hypermethylation in normal development is also secondary to prior silencing through other mechanisms.
- Several hypotheses now exist as to the impact of CGI hypermethylation on carcinogenesis and progression. The occasional hypermethylation of rare driver genes might directly promote carcinogenesis. Widespread CGI hypermethylation could also result in more aggressive cancers by blocking cellular differentiation or act as a protective mechanism hindering progression by preventing epigenetic adaptation to changing conditions.
- The mechanism underpinning aberrant CpG island hypermethylation remains elusive but genome-scale studies have refined our hypotheses and demonstrated that a distinct gene set is affected.

## FUNDING

Work in R.M.’s lab is supported by the Medical Research Council, the Biotechnology and Biological Sciences Research Council and by the Innovative Medicine Initiative Joint Undertaking (IMI JU) under grant agreement number 115 001 (MARCAR project). D.S. is supported by the Medical Research Council, Breakthrough Breast Cancer and Breast Cancer Campaign.

## References

[1] Bird A (2007). Perceptions of epigenetics. Nature.

[2] You JS, Jones PA (2012). Cancer genetics and epigenetics: two sides of the same coin?. Cancer Cell.

[3] Ziller MJ, Muller F, Liao J (2011). Genomic distribution and inter-sample variation of non-CpG methylation across human cell types. PLoS Genet.

[4] Suzuki MM, Bird A (2008). DNA methylation landscapes: provocative insights from epigenomics. Nat Rev Genet.

[5] Illingworth RS, Gruenewald-Schneider U, Webb S (2010). Orphan CpG islands identify numerous conserved promoters in the mammalian genome. PLoS Genet.

[6] Bird AP (1980). DNA methylation and the frequency of CpG in animal DNA. Nucleic Acids Res.

[7] Rideout WM, Coetzee GA, Olumi AF (1990). 5-Methylcytosine as an endogenous mutagen in the human LDL receptor and p53 genes. Science.

[8] Fournier A, Sasai N, Nakao M (2012). The role of methyl-binding proteins in chromatin organization and epigenome maintenance. Brief Funct Genomics.

[9] Cedar H, Bergman Y (2012). Programming of DNA methylation patterns. Annu Rev Biochem.

[10] Bock C, Beerman I, Lien WH (2012). DNA methylation dynamics during in vivo differentiation of blood and skin stem cells. Mol Cell.

[11] Deaton AM, Webb S, Kerr AR (2011). Cell type-specific DNA methylation at intragenic CpG islands in the immune system. Genome Res.

[12] Irizarry RA, Ladd-Acosta C, Wen B (2009). The human colon cancer methylome shows similar hypo- and hypermethylation at conserved tissue-specific CpG island shores. Nat Genet.

[13] Ji H, Ehrlich LI, Seita J (2010). Comprehensive methylome map of lineage commitment from haematopoietic progenitors. Nature.

[14] Kriaucionis S, Heintz N (2009). The nuclear DNA base 5-hydroxymethylcytosine is present in Purkinje neurons and the brain. Science.

[15] Nestor CE, Ottaviano R, Reddington J (2012). Tissue-type is a major modifier of the 5-hydroxymethylcytosine content of human genes. Genome Res.

[16] Ehrlich M (2009). DNA hypomethylation in cancer cells. Epigenomics.

[17] Wild L, Flanagan JM (2010). Genome-wide hypomethylation in cancer may be a passive consequence of transformation. Biochim Biophys Acta.

[18] Berman BP, Weisenberger DJ, Aman JF (2011). Regions of focal DNA hypermethylation and long-range hypomethylation in colorectal cancer coincide with nuclear lamina-associated domains. Nat Genet.

[19] Hon GC, Hawkins RD, Caballero OL (2012). Global DNA hypomethylation coupled to repressive chromatin domain formation and gene silencing in breast cancer. Genome Res.

[20] Haffner MC, Chaux A, Meeker AK (2011). Global 5-hydroxymethylcytosine content is significantly reduced in tissue stem/progenitor cell compartments and in human cancers. Oncotarget.

[21] Jin SG, Jiang Y, Qiu R (2011). 5-Hydroxymethylcytosine is strongly depleted in human cancers but its levels do not correlate with IDH1 mutations. Cancer Res.

[22] Lian CG, Xu Y, Ceol C (2012). Loss of 5-hydroxymethylcytosine is an epigenetic hallmark of melanoma. Cell.

[23] Boyes J, Bird A (1992). Repression of genes by DNA methylation depends on CpG density and promoter strength: evidence for involvement of a methyl-CpG binding protein. EMBO J.

[24] Feinberg AP, Tycko B (2004). The history of cancer epigenetics. Nat Rev Cancer.

[25] Weinberg RA (2007). The Biology of Cancer.

[26] Herman JG, Baylin SB (2003). Gene silencing in cancer in association with promoter hypermethylation. N Engl J Med.

[27] Greger V, Debus N, Lohmann D (1994). Frequency and parental origin of hypermethylated RB1 alleles in retinoblastoma. Hum Genet.

[28] Greger V, Passarge E, Hopping W (1989). Epigenetic changes may contribute to the formation and spontaneous regression of retinoblastoma. Hum Genet.

[29] Ohtani-Fujita N, Fujita T, Aoike A (1993). CpG methylation inactivates the promoter activity of the human retinoblastoma tumor-suppressor gene. Oncogene.

[30] Sakai T, Toguchida J, Ohtani N (1991). Allele-specific hypermethylation of the retinoblastoma tumor-suppressor gene. Am J Hum Genet.

[31] Cunningham JM, Christensen ER, Tester DJ (1998). Hypermethylation of the hMLH1 promoter in colon cancer with microsatellite instability. Cancer Res.

[32] Herman JG, Umar A, Polyak K (1998). Incidence and functional consequences of hMLH1 promoter hypermethylation in colorectal carcinoma. Proc Natl Acad Sci USA.

[33] Esteller M, Silva JM, Dominguez G (2000). Promoter hypermethylation and BRCA1 inactivation in sporadic breast and ovarian tumors. J Natl Cancer Inst.

[34] Vogelstein B, Kinzler KW (2004). Cancer genes and the pathways they control. Nat Med.

[35] Esteller M, Fraga MF, Guo M (2001). DNA methylation patterns in hereditary human cancers mimic sporadic tumorigenesis. Hum Mol Genet.

[36] Myohanen SK, Baylin SB, Herman JG (1998). Hypermethylation can selectively silence individual p16ink4A alleles in neoplasia. Cancer Res.

[37] Ohtani-Fujita N, Dryja TP, Rapaport JM (1997). Hypermethylation in the retinoblastoma gene is associated with unilateral, sporadic retinoblastoma. Cancer Genet Cytogenet.

[38] Dworkin AM, Spearman AD, Tseng SY (2009). Methylation not a frequent “second hit” in tumors with germline BRCA mutations. Fam Cancer.

[39] Tung N, Miron A, Schnitt SJ (2010). Prevalence and predictors of loss of wild type BRCA1 in estrogen receptor positive and negative BRCA1-associated breast cancers. Breast Cancer Res.

[40] Sproul D, Kitchen RR, Nestor CE (2012). Tissue of origin determines cancer-associated CpG island promoter hypermethylation patterns. Genome Biol.

[41] Herman JG, Latif F, Weng Y (1994). Silencing of the VHL tumor-suppressor gene by DNA methylation in renal carcinoma. Proc Natl Acad Sci USA.

[42] Hitchins MP, Rapkins RW, Kwok CT (2011). Dominantly inherited constitutional epigenetic silencing of MLH1 in a cancer-affected family is linked to a single nucleotide variant within the 5′UTR. Cancer Cell.

[43] Ligtenberg MJ, Kuiper RP, Chan TL (2009). Heritable somatic methylation and inactivation of MSH2 in families with Lynch syndrome due to deletion of the 3′ exons of TACSTD1. Nat Genet.

[44] Turner N, Tutt A, Ashworth A (2004). Hallmarks of ‘BRCAness’ in sporadic cancers. Nat Rev Cancer.

[45] Stratton MR (1997). Pathology of familial breast cancer: differences between breast cancers in carriers of BRCA1 or BRCA2 mutations and sporadic cases. Breast Cancer Linkage Consortium*. Lancet*.

[46] Weigelt B, Geyer FC, Reis-Filho JS (2010). Histological types of breast cancer: how special are they?. Mol Oncol.

[47] Turner NC, Reis-Filho JS, Russell AM (2007). BRCA1 dysfunction in sporadic basal-like breast cancer. Oncogene.

[48] Matros E, Wang ZC, Lodeiro G (2005). BRCA1 promoter methylation in sporadic breast tumors: relationship to gene expression profiles. Breast Cancer Res Treat.

[49] TCGA (2011). Integrated genomic analyses of ovarian carcinoma. Nature.

[50] Hammerman PS, Hayes DN, Wilkerson MD (2012). Comprehensive genomic characterization of squamous cell lung cancers. Nature.

[51] Patel K, Dickson J, Din S (2010). Targeting of 5-aza-2′-deoxycytidine residues by chromatin-associated DNMT1 induces proteasomal degradation of the free enzyme. Nucleic Acids Res.

[52] Veigl ML, Kasturi L, Olechnowicz J (1998). Biallelic inactivation of hMLH1 by epigenetic gene silencing, a novel mechanism causing human MSI cancers. Proc Natl Acad Sci USA.

[53] Veeck J, Ropero S, Setien F (2010). BRCA1 CpG island hypermethylation predicts sensitivity to poly(adenosine diphosphate)-ribose polymerase inhibitors. J Clin Oncol.

[54] Robert MF, Morin S, Beaulieu N (2003). DNMT1 is required to maintain CpG methylation and aberrant gene silencing in human cancer cells. Nat Genet.

[55] Rhee I, Bachman KE, Park BH (2002). DNMT1 and DNMT3b cooperate to silence genes in human cancer cells. Nature.

[56] Mohandas T, Sparkes RS, Shapiro LJ (1981). Reactivation of an inactive human X chromosome: evidence for X inactivation by DNA methylation. Science.

[57] Lock LF, Takagi N, Martin GR (1987). Methylation of the Hprt gene on the inactive X occurs after chromosome inactivation. Cell.

[58] Okamoto I, Heard E (2009). Lessons from comparative analysis of X-chromosome inactivation in mammals. Chromosome Res.

[59] Sado T, Fenner MH, Tan SS (2000). X inactivation in the mouse embryo deficient for Dnmt1: distinct effect of hypomethylation on imprinted and random X inactivation. Dev Biol.

[60] Sado T, Okano M, Li E (2004). De novo DNA methylation is dispensable for the initiation and propagation of X chromosome inactivation. Development.

[61] Bestor TH (2003). Unanswered questions about the role of promoter methylation in carcinogenesis. Ann N Y Acad Sci.

[62] Clark SJ, Melki J (2002). DNA methylation and gene silencing in cancer: which is the guilty party?. Oncogene.

[63] Deaton AM, Bird A (2011). CpG islands and the regulation of transcription. Genes Dev.

[64] Fearon ER (2000). BRCA1 and E-cadherin promoter hypermethylation and gene inactivation in cancer-association or mechanism?. J Natl Cancer Inst.

[65] Levanon D, Bernstein Y, Negreanu V (2011). Absence of Runx3 expression in normal gastrointestinal epithelium calls into question its tumour suppressor function. EMBO Mol Med.

[66] Baylin SB, Hoppener JW, de Bustros A (1986). DNA methylation patterns of the calcitonin gene in human lung cancers and lymphomas. Cancer Res.

[67] Costello JF, Fruhwald MC, Smiraglia DJ (2000). Aberrant CpG-island methylation has non-random and tumour-type-specific patterns. Nat Genet.

[68] Toyota M, Ahuja N, Ohe-Toyota M (1999). CpG island methylator phenotype in colorectal cancer. Proc Natl Acad Sci USA.

[69] Fang F, Turcan S, Rimner A (2011). Breast cancer methylomes establish an epigenomic foundation for metastasis. Sci Transl Med.

[70] Figueroa ME, Abdel-Wahab O, Lu C (2010). Leukemic IDH1 and IDH2 mutations result in a hypermethylation phenotype, disrupt TET2 function, and impair hematopoietic differentiation. Cancer Cell.

[71] Noushmehr H, Weisenberger DJ, Diefes K (2010). Identification of a CpG island methylator phenotype that defines a distinct subgroup of glioma. Cancer Cell.

[72] Teodoridis JM, Hardie C, Brown R (2008). CpG island methylator phenotype (CIMP) in cancer: causes and implications. Cancer Lett.

[73] Welch JS, Ley TJ, Link DC (2012). The origin and evolution of mutations in acute myeloid leukemia. Cell.

[74] Antequera F, Boyes J, Bird A (1990). High levels of de novo methylation and altered chromatin structure at CpG islands in cell lines. Cell.

[75] Sproul D, Nestor C, Culley J (2011). Transcriptionally repressed genes become aberrantly methylated and distinguish tumors of different lineages in breast cancer. Proc Natl Acad Sci USA.

[76] Hinoue T, Weisenberger DJ, Lange CP (2012). Genome-scale analysis of aberrant DNA methylation in colorectal cancer. Genome Res.

[77] Houshdaran S, Hawley S, Palmer C (2010). DNA methylation profiles of ovarian epithelial carcinoma tumors and cell lines. PLoS One.

[78] Keshet I, Schlesinger Y, Farkash S (2006). Evidence for an instructive mechanism of de novo methylation in cancer cells. Nat Genet.

[79] O'Riain C, O'Shea DM, Yang Y (2009). Array-based DNA methylation profiling in follicular lymphoma. Leukemia.

[80] Pike BL, Greiner TC, Wang X (2008). DNA methylation profiles in diffuse large B-cell lymphoma and their relationship to gene expression status. Leukemia.

[81] Wolff EM, Chihara Y, Pan F (2010). Unique DNA methylation patterns distinguish noninvasive and invasive urothelial cancers and establish an epigenetic field defect in premalignant tissue. Cancer Res.

[82] Easwaran H, Johnstone SE, Van Neste L (2012). A DNA hypermethylation module for the stem/progenitor cell signature of cancer. Genome Res.

[83] Bird A (2002). DNA methylation patterns and epigenetic memory. Genes Dev.

[84] Jones PA (2012). Functions of DNA methylation: islands, start sites, gene bodies and beyond. Nat Rev Genet.

[85] Sato S, Yoshida W, Soejima H (2011). Methylation dynamics of IG-DMR and Gtl2-DMR during murine embryonic and placental development. Genomics.

[86] Epsztejn-Litman S, Feldman N, Abu-Remaileh M (2008). De novo DNA methylation promoted by G9a prevents reprogramming of embryonically silenced genes. Nat Struct Mol Biol.

[87] Feldman N, Gerson A, Fang J (2006). G9a-mediated irreversible epigenetic inactivation of Oct-3/4 during early embryogenesis. Nat Cell Biol.

[88] Mohn F, Weber M, Rebhan M (2008). Lineage-specific polycomb targets and de novo DNA methylation define restriction and potential of neuronal progenitors. Mol Cell.

[89] Mutskov V, Felsenfeld G (2004). Silencing of transgene transcription precedes methylation of promoter DNA and histone H3 lysine 9. EMBO J.

[90] Ruiz S, Diep D, Gore A (2012). Identification of a specific reprogramming-associated epigenetic signature in human induced pluripotent stem cells. Proc Natl Acad Sci USA.

[91] Mikkelsen TS, Hanna J, Zhang X (2008). Dissecting direct reprogramming through integrative genomic analysis. Nature.

[92] Baylin SB, Jones PA (2011). A decade of exploring the cancer epigenome—biological and translational implications. Nat Rev Cancer.

[93] Kalari S, Pfeifer GP (2010). Identification of driver and passenger DNA methylation in cancer by epigenomic analysis. Adv Genet.

[94] Stratton MR, Campbell PJ, Futreal PA (2009). The cancer genome. Nature.

[95] Gilbertson RJ, Graham TA (2012). Cancer: resolving the stem-cell debate. Nature.

[96] Hosoya K, Yamashita S, Ando T (2009). Adenomatous polyposis coli 1A is likely to be methylated as a passenger in human gastric carcinogenesis. Cancer Lett.

[97] Kodzius R, Kojima M, Nishiyori H (2006). CAGE: cap analysis of gene expression. Nat Methods.

[98] Wu ZQ, Li XY, Hu CY (2012). Canonical Wnt signaling regulates Slug activity and links epithelial-mesenchymal transition with epigenetic Breast Cancer 1, Early Onset (BRCA1) repression. Proc Natl Acad Sci USA.

[99] Esteller M, Corn PG, Baylin SB (2001). A gene hypermethylation profile of human cancer. Cancer Res.

[100] Gil J, Peters G (2006). Regulation of the INK4b-ARF-INK4a tumour suppressor locus: all for one or one for all. Nat Rev Mol Cell Biol.

[101] Bracken AP, Kleine-Kohlbrecher D, Dietrich N (2007). The Polycomb group proteins bind throughout the INK4A-ARF locus and are disassociated in senescent cells. Genes Dev.

[102] Jacobs JJ, Kieboom K, Marino S (1999). The oncogene and Polycomb-group gene bmi-1 regulates cell proliferation and senescence through the ink4a locus. Nature.

[103] Bachman KE, Park BH, Rhee I (2003). Histone modifications and silencing prior to DNA methylation of a tumor suppressor gene. Cancer Cell.

[104] Hinshelwood RA, Melki JR, Huschtscha LI (2009). Aberrant de novo methylation of the p16INK4A CpG island is initiated post gene silencing in association with chromatin remodelling and mimics nucleosome positioning. Hum Mol Genet.

[105] Gendrel AV, Apedaile A, Coker H (2012). Smchd1-dependent and -independent pathways determine developmental dynamics of CpG island methylation on the inactive x chromosome. Dev Cell.

[106] De Carvalho DD, Sharma S, You JS (2012). DNA methylation screening identifies driver epigenetic events of cancer cell survival. Cancer Cell.

[107] Ogino S, Nosho K, Kirkner GJ (2009). CpG island methylator phenotype, microsatellite instability, BRAF mutation and clinical outcome in colon cancer. Gut.

[108] Wood LD, Parsons DW, Jones S (2007). The genomic landscapes of human breast and colorectal cancers. Science.

[109] Ohm JE, McGarvey KM, Yu X (2007). A stem cell-like chromatin pattern may predispose tumor suppressor genes to DNA hypermethylation and heritable silencing. Nat Genet.

[110] Schlesinger Y, Straussman R, Keshet I (2007). Polycomb-mediated methylation on Lys27 of histone H3 pre-marks genes for de novo methylation in cancer. Nat Genet.

[111] Widschwendter M, Fiegl H, Egle D (2007). Epigenetic stem cell signature in cancer. Nat Genet.

[112] Ben-Porath I, Thomson MW, Carey VJ (2008). An embryonic stem cell-like gene expression signature in poorly differentiated aggressive human tumors. Nat Genet.

[113] Kim J, Woo AJ, Chu J (2010). A Myc network accounts for similarities between embryonic stem and cancer cell transcription programs. Cell.

[114] Bracken AP, Dietrich N, Pasini D (2006). Genome-wide mapping of Polycomb target genes unravels their roles in cell fate transitions. Genes Dev.

[115] Helman E, Naxerova K, Kohane IS (2012). DNA hypermethylation in lung cancer is targeted at differentiation-associated genes. Oncogene.

[116] Sasaki M, Knobbe CB, Munger JC (2012). IDH1(R132H) mutation increases murine haematopoietic progenitors and alters epigenetics. Nature.

[117] Sasaki M, Knobbe CB, Itsumi M (2012). D-2-hydroxyglutarate produced by mutant IDH1 perturbs collagen maturation and basement membrane function. Genes Dev.

[118] Xu W, Yang H, Liu Y (2011). Oncometabolite 2-hydroxyglutarate is a competitive inhibitor of alpha-ketoglutarate-dependent dioxygenases. Cancer Cell.

[119] Nguyen DX, Bos PD, Massague J (2009). Metastasis: from dissemination to organ-specific colonization. Nat Rev Cancer.

[120] Lord CJ, Ashworth A (2012). The DNA damage response and cancer therapy. Nature.

[121] McDermott U, Downing JR, Stratton MR (2011). Genomics and the continuum of cancer care. N Engl J Med.

[122] Adams PD (2007). Remodeling of chromatin structure in senescent cells and its potential impact on tumor suppression and aging. Gene.

[123] Feinberg AP, Ohlsson R, Henikoff S (2006). The epigenetic progenitor origin of human cancer. Nat Rev Genet.

[124] Issa JP, Vertino PM, Wu J (1993). Increased cytosine DNA-methyltransferase activity during colon cancer progression. J Natl Cancer Inst.

[125] el-Deiry WS, Nelkin BD, Celano P (1991). High expression of the DNA methyltransferase gene characterizes human neoplastic cells and progression stages of colon cancer. Proc Natl Acad Sci USA.

[126] Eads CA, Danenberg KD, Kawakami K (1999). CpG island hypermethylation in human colorectal tumors is not associated with DNA methyltransferase overexpression. Cancer Res.

[127] Robertson KD, Keyomarsi K, Gonzales FA (2000). Differential mRNA expression of the human DNA methyltransferases (DNMTs) 1, 3a and 3b during the G(0)/G(1) to S phase transition in normal and tumor cells. Nucleic Acids Res.

[128] Ibrahim AE, Arends MJ, Silva AL (2011). Sequential DNA methylation changes are associated with DNMT3B overexpression in colorectal neoplastic progression. Gut.

[129] Laird PW, Jackson-Grusby L, Fazeli A (1995). Suppression of intestinal neoplasia by DNA hypomethylation. Cell.

[130] Lin H, Yamada Y, Nguyen S (2006). Suppression of intestinal neoplasia by deletion of Dnmt3b. Mol Cell Biol.

[131] Linhart HG, Lin H, Yamada Y (2007). Dnmt3b promotes tumorigenesis in vivo by gene-specific de novo methylation and transcriptional silencing. Genes Dev.

[132] Steine EJ, Ehrich M, Bell GW (2011). Genes methylated by DNA methyltransferase 3b are similar in mouse intestine and human colon cancer. J Clin Invest.

[133] Ley TJ, Ding L, Walter MJ (2010). DNMT3A mutations in acute myeloid leukemia. N Engl J Med.

[134] Walter MJ, Ding L, Shen D (2011). Recurrent DNMT3A mutations in patients with myelodysplastic syndromes. Leukemia.

[135] Yan XJ, Xu J, Gu ZH (2011). Exome sequencing identifies somatic mutations of DNA methyltransferase gene DNMT3A in acute monocytic leukemia. Nat Genet.

[136] Ribeiro AF, Pratcorona M, Erpelinck-Verschueren C (2012). Mutant DNMT3A: a marker of poor prognosis in acute myeloid leukemia. Blood.

[137] Shah MY, Vasanthakumar A, Barnes NY (2010). DNMT3B7, a truncated DNMT3B isoform expressed in human tumors, disrupts embryonic development and accelerates lymphomagenesis. Cancer Res.

[138] Goll MG, Bestor TH (2005). Eukaryotic cytosine methyltransferases. Annu Rev Biochem.

[139] Van Emburgh BO, Robertson KD (2011). Modulation of Dnmt3b function in vitro by interactions with Dnmt3L, Dnmt3a and Dnmt3b splice variants. Nucleic Acids Res.

[140] Prokhortchouk A, Sansom O, Selfridge J (2006). Kaiso-deficient mice show resistance to intestinal cancer. Mol Cell Biol.

[141] Sansom OJ, Berger J, Bishop SM (2003). Deficiency of Mbd2 suppresses intestinal tumorigenesis. Nat Genet.

[142] Vire E, Brenner C, Deplus R (2006). The Polycomb group protein EZH2 directly controls DNA methylation. Nature.

[143] Denis H, Ndlovu MN, Fuks F (2011). Regulation of mammalian DNA methyltransferases: a route to new mechanisms. EMBO Rep.

[144] Rush M, Appanah R, Lee S (2009). Targeting of EZH2 to a defined genomic site is sufficient for recruitment of Dnmt3a but not de novo DNA methylation. Epigenetics.

[145] Fouse SD, Shen Y, Pellegrini M (2008). Promoter CpG methylation contributes to ES cell gene regulation in parallel with Oct4/Nanog, PcG complex, and histone H3 K4/K27 trimethylation. Cell Stem Cell.

[146] Kondo Y, Shen L, Cheng AS (2008). Gene silencing in cancer by histone H3 lysine 27 trimethylation independent of promoter DNA methylation. Nat Genet.

[147] Statham AL, Robinson MD, Song JZ (2012). Bisulfite sequencing of chromatin immunoprecipitated DNA (BisChIP-seq) directly informs methylation status of histone-modified DNA. Genome Res.

[148] Brinkman AB, Gu H, Bartels SJ (2012). Sequential ChIP-bisulfite sequencing enables direct genome-scale investigation of chromatin and DNA methylation cross-talk. Genome Res.

[149] Lynch MD, Smith AJ, De Gobbi M (2012). An interspecies analysis reveals a key role for unmethylated CpG dinucleotides in vertebrate Polycomb complex recruitment. EMBO J.

[150] Gal-Yam EN, Egger G, Iniguez L (2008). Frequent switching of Polycomb repressive marks and DNA hypermethylation in the PC3 prostate cancer cell line. Proc Natl Acad Sci USA.

[151] Meissner A, Mikkelsen TS, Gu H (2008). Genome-scale DNA methylation maps of pluripotent and differentiated cells. Nature.

[152] Thomson JP, Skene PJ, Selfridge J (2010). CpG islands influence chromatin structure via the CpG-binding protein Cfp1. Nature.

[153] Ooi SK, Qiu C, Bernstein E (2007). DNMT3L connects unmethylated lysine 4 of histone H3 to de novo methylation of DNA. Nature.

[154] Clouaire T, Webb S, Skene P (2012). Cfp1 integrates both CpG content and gene activity for accurate H3K4me3 deposition in embryonic stem cells. Genes Dev.

[155] Zilberman D, Coleman-Derr D, Ballinger T (2008). Histone H2A.Z and DNA methylation are mutually antagonistic chromatin marks. Nature.

[156] Zemach A, McDaniel IE, Silva P (2010). Genome-wide evolutionary analysis of eukaryotic DNA methylation. Science.

[157] Conerly ML, Teves SS, Diolaiti D (2010). Changes in H2A.Z occupancy and DNA methylation during B-cell lymphomagenesis. Genome Res.

[158] Franchini DM, Schmitz KM, Petersen-Mahrt SK (2012). 5-methylcytosine DNA demethylation: more than losing a methyl group. Annu Rev Genet.

[159] Tahiliani M, Koh KP, Shen Y (2009). Conversion of 5-methylcytosine to 5-hydroxymethylcytosine in mammalian DNA by MLL partner TET1. Science.

[160] Williams K, Christensen J, Pedersen MT (2011). TET1 and hydroxymethylcytosine in transcription and DNA methylation fidelity. Nature.

[161] Wu H, D’Alessio AC, Ito S (2011). Dual functions of Tet1 in transcriptional regulation in mouse embryonic stem cells. Nature.

[162] Williams K, Christensen J, Helin K (2012). DNA methylation: TET proteins-guardians of CpG islands?. EMBO Rep.

[163] Abdel-Wahab O, Mullally A, Hedvat C (2009). Genetic characterization of TET1, TET2, and TET3 alterations in myeloid malignancies. Blood.

[164] Gertz J, Varley KE, Reddy TE (2011). Analysis of DNA methylation in a three-generation family reveals widespread genetic influence on epigenetic regulation. PLoS Genet.

[165] Lienert F, Wirbelauer C, Som I (2011). Identification of genetic elements that autonomously determine DNA methylation states. Nat Genet.

[166] Thurman RE, Rynes E, Humbert R (2012). The accessible chromatin landscape of the human genome. Nature.

[167] Hervouet E, Vallette FM, Cartron PF (2009). Dnmt3/transcription factor interactions as crucial players in targeted DNA methylation. Epigenetics.

[168] Di Croce L, Raker VA, Corsaro M (2002). Methyltransferase recruitment and DNA hypermethylation of target promoters by an oncogenic transcription factor. Science.

[169] Feltus FA, Lee EK, Costello JF (2003). Predicting aberrant CpG island methylation. Proc Natl Acad Sci USA.

[170] Gebhard C, Benner C, Ehrich M (2010). General transcription factor binding at CpG islands in normal cells correlates with resistance to de novo DNA methylation in cancer cells. Cancer Res.

[171] Takeshima H, Yamashita S, Shimazu T (2009). The presence of RNA polymerase II, active or stalled, predicts epigenetic fate of promoter CpG islands. Genome Res.

[172] Boumber YA, Kondo Y, Chen X (2008). An Sp1/Sp3 binding polymorphism confers methylation protection. PLoS Genet.

[173] Brookes E, de Santiago I, Hebenstreit D (2012). Polycomb associates genome-wide with a specific RNA polymerase II variant, and regulates metabolic genes in ESCs. Cell Stem Cell.

[174] Enderle D, Beisel C, Stadler MB (2011). Polycomb preferentially targets stalled promoters of coding and noncoding transcripts. Genome Res.

[175] Holm K, Hegardt C, Staaf J (2010). Molecular subtypes of breast cancer are associated with characteristic DNA methylation patterns. Breast Cancer Res.

[176] Bernstein BE, Mikkelsen TS, Xie X (2006). A bivalent chromatin structure marks key developmental genes in embryonic stem cells. Cell.

[177] De Gobbi M, Garrick D, Lynch M (2011). Generation of bivalent chromatin domains during cell fate decisions. Epigenetics Chromatin.

[178] Estecio MR, Gallegos J, Vallot C (2010). Genome architecture marked by retrotransposons modulates predisposition to DNA methylation in cancer. Genome Res.

[179] Ohm JE, Baylin SB (2007). Stem cell chromatin patterns: an instructive mechanism for DNA hypermethylation?. Cell Cycle.

[180] Landan G, Cohen NM, Mukamel Z (2012). Epigenetic polymorphism and the stochastic formation of differentially methylated regions in normal and cancerous tissues. Nat Genet.

[181] Rakyan VK, Down TA, Balding DJ (2011). Epigenome-wide association studies for common human diseases. Nat Rev Genet.

[182] Heyn H, Esteller M (2012). DNA methylation profiling in the clinic: applications and challenges. Nat Rev Genet.

[183] Vanharanta S, Shu W, Brenet F (2013). Epigenetic expansion of VHL-HIF signal output drives multiorgan metastasis in renal cancer. Nat Med.

